# Detection and Isolation of Airborne Influenza A H3N2 Virus Using a Sioutas Personal Cascade Impactor Sampler

**DOI:** 10.1155/2013/656825

**Published:** 2013-10-10

**Authors:** John A. Lednicky, Julia C. Loeb

**Affiliations:** ^1^Department of Environmental and Global Health, College of Public Health and Health Professions, P.O. Box 100188, Gainesville, FL 32610, USA; ^2^Emerging Pathogens Institute, University of Florida, Gainesville, FL 32610, USA

## Abstract

The air we breathe contains microorganisms that can cause infectious respiratory diseases. After two occupants of an apartment were diagnosed with influenza in February of 2013, efforts were made to detect and isolate airborne influenza virus using two different types of active air samplers: a Sioutas Personal Cascade Impactor Sampler (PCIS) and an SKC BioSampler. The PCIS collects size-fractionated particles by impaction on polytetrafluoroethylene filters, whereas the SKC BioSampler collects airborne particles in liquid media. Influenza H3N2 virus was collected by both types of air samplers. The PCIS collected a range of particle sizes containing influenza virus near one of the sick individuals but only ultrafine particles when the samplers were positioned farther away. Viable virus was present in the liquid collection media of the SKC BioSampler and some PCIS filters. These findings suggest that influenza patients produce ultrafine aerosol particles that contain viable virus.

## 1. Introduction

Airborne microorganisms are typically present in the air we breathe and are potential causes of infectious diseases. Influenza viruses are among the respiratory pathogens that can be transmitted by airborne routes [1–4]. Breathing, coughing, sneezing, and talking during the course of influenza generate a cloud of airborne particles containing influenza virus. The airborne particles have diameters that range from a few millimeters (large droplets formed during coughing and sneezing) to submicron (<1 *μ*m) (formed during breathing). Small particles (≤5 *μ*m) including droplet nuclei from evaporated larger particles can remain airborne for hours (these concepts and their relevance to influenza viruses are discussed in [1, 3–14]). Presently, the US Centers for Disease Control and Prevention (CDC) and the World Health Organization (WHO) state that influenza virus transmission occurs mostly by large-particle respiratory droplets that travel only a short distance from the source (1.829 m (6 feet) according to the CDC and 1 m (3.3 feet) according to the WHO) [15–17]. Noteworthy, deposition of influenza virus into the lungs (as small particles) versus the upper respiratory tract (as large droplets) may increase infection risk and illness severity [3, 9, 18–20].

 Assessments of microbiological air quality are useful for studies of the airborne transmission of pathogens and of other biological particles in the air that may cause noninfectious diseases (such as allergy to pollen). The two principle methods of assessing microbiological air quality are passive monitoring and active sampling [21]. Both methods were designed for studies of airborne bacteria or fungi. Passive monitoring is typically performed using settle plates, which are opened and exposed to the air for a given time, then incubated and resulting bacterial and fungal colonies are analyzed. Active sampling is performed with a microbiological air sampler that mechanically draws a known volume of air over, or through, a particle collection device. The particles are subsequently removed from the collection medium and analyzed. Air samplers are also used to collect airborne viruses [22], but it is difficult to do so primarily due to their small size and relatively low concentration in ambient air [23]. Also, most of the available bioaerosol samplers are not suitable for the collection of viruses for reasons that include (a) they typically run for short periods of time (min), making it difficult to capture large volumes, (b) most do not separate particles by size, and (c), they are inefficient at collecting submicron particles [23–25]. 

**Figure 1:**
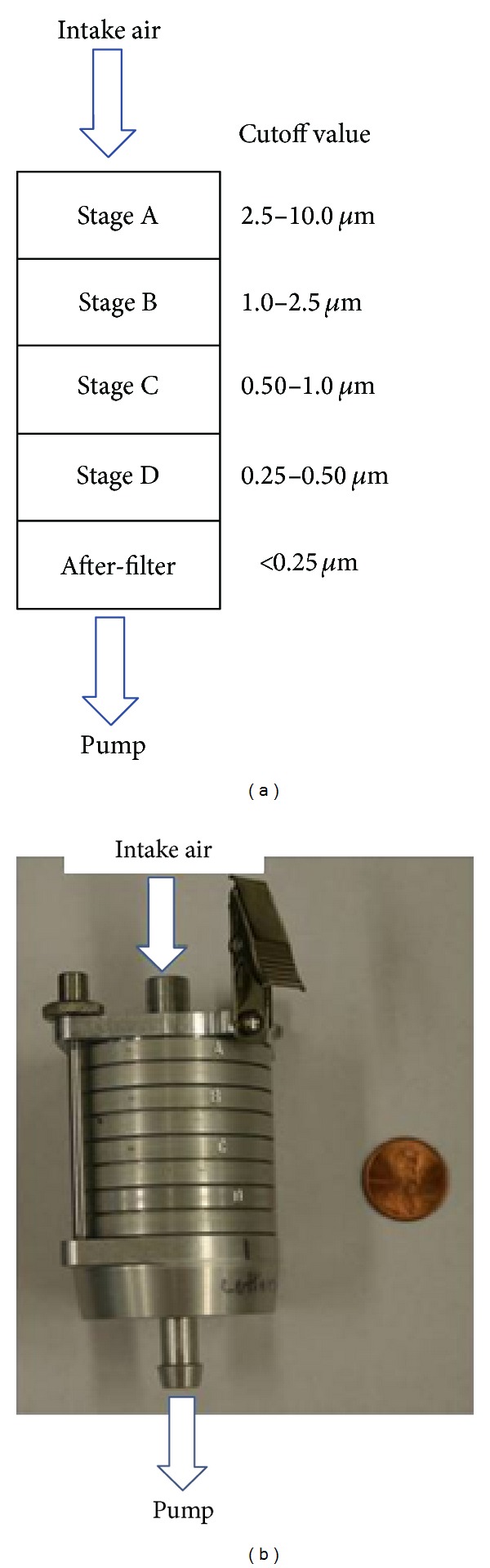
Sioutas Personal Cascade Impactor Sampler (PCIS). (a) Schematic representation of the five interior sections of a PCIS. Each stage has an upper acceleration compartment and a lower impaction-collector surface. (b) Photograph of a PCIS unit. For size perspective, a coin is shown to the right of the PCIS.

 The PCIS [26] separates airborne particles in a cascading fashion and simultaneously collects the size-fractionated particles by impaction on polytetrafluoroethylene (PTFE) filters. It has collection filters on four impaction stages (A–D), and optionally, an after-filter can be added onto a 5th stage. The PCIS is designed to separate and collect airborne particulate matter above the cutpoint of five size ranges: >2.5 *μ*m (Stage A), 1.0 to 2.5 *μ*m (Stage B), 0.50 to 1.0 *μ*m (Stage C), 0.25 to 0.50 *μ*m (Stage D), and <0.25 *μ*m (collected on an after-filter) (Figure 1). PTFE filters, better known as Teflon filters, can collect particles at high efficiency above the cutpoints without the need for coatings [26]. Coatings can also reduce the recovery efficiency of viable virus. Lastly, for the efficient collection of airborne influenza virus, the filters are not prewetted with methanol prior to use [24]. 

The SKC BioSampler collects unfractionated airborne particles in liquid media [27]. Made out of glass, the SKC BioSampler is purpose-designed to collect particles of a size range that would go through human nasal passages. The SKC BioSampler has three 0.630 mm tangential sonic nozzles; air passing through the nozzles creates a swirling flow of collection medium during sampling. Compared to other impingers, particle skip and damage to the collected microbial agent are potentially minimized by the collection swirling liquid. Whereas the SKC BioSampler has worked well for the collection of airborne bacteria and fungi in the field, it is nevertheless inefficient at recovering submicrometer and ultrafine virus aerosols, with collection efficiencies of <10% for particles in the 30 to 100 nm size range [28]. Nevertheless, mostly because significantly better alternative devices are not readily available, the SKC BioSampler has been used for the collection of airborne influenza virus [23, 29–31].

An adult and a child living in an apartment in Gainesville, Florida, were diagnosed with influenza caused by type A influenza virus. As both occupants had been vaccinated against influenza A and B viruses in November of 2012, there was a possibility that they were infected with a new influenza virus strain significantly different than from those in the vaccines. We (opportunistically) evaluated airborne material from the apartment that had been collected on PCIS filters and in SKC BioSampler liquid collection media for the presence of viable influenza A virus and/or viral genomic RNA. 

## 2. Materials and Methods

### 2.1. Study Site

The dwelling that was evaluated in this report is a single-family four-bedroom apartment in Gainesville, Florida, USA, that has its own heating, ventilation, and air conditioning (HVAC) system. Passive air exchange with the outside was minimal, as windows and an entry door were kept closed due to cold weather.

### 2.2. Metrology

Relative humidity (RH) and temperature (*T*) were measured using an electronic NIST traceable RH and *T* meter (ThermoFisher, catalog number 11-661-19).

### 2.3. Laboratory

Virology work was performed in a biosafety level 2-enhanced laboratory at the University of Florida.

### 2.4. SKC BioSamplers

The SKC (SKC, Inc., Eighty Four, PA, USA) BioSampler (SKC catalog number 225-9595) is a widely-used device for microbiological air quality assessments. SKC BioSamplers were operated with 115 volt Vac-U-Go sampling pumps (SKC, Inc., catalog number 228-9605). The pumps were warmed up by running them for 5 min prior to use. To protect the pumps against moisture, in-line vapor traps (SKC Inc., catalog number 225-22-01) were connected between the SKC BioSamplers and sampling pumps. SKC BioSamplers were sterilized by autoclaving prior to use then filled with 15 mL of sterile phosphate buffered saline with calcium and magnesium (PBS), 0.5% w/v purified BSA fraction V (Life Technologies, Grand Island, NY, USA), and 0.25% (v/v) molecular-grade antifoam agent B (Sigma-Aldrich, Inc., St. Louis, MO, USA) for virus collection [9]. The operating pressure was measured using the pump's built-in manometer (pressure gauge); the sampling flow rate was 12.5 L/min, determined using a flow meter attached to the inlet of the SKC BioSampler prior to use. This suggested that the SKC BioSampler was operating normally, because at normal atmospheric conditions, the flow rate should always be around 12.5 L/min when there is a pressure drop of at least 0.5 atm. For each unit, a collection time of 30 min was used to sample approximately 375 liters (0.375 m^3^) of air.

### 2.5. Sioutas Personal Cascade Impactor Sampler (PCIS)

The PCIS (SKC, Inc., catalog number 225-370) units were used with Leland Legacy pumps (SKC, Inc., cat number 100-3002) and operated at a flow rate of 9 L/min. Polytetrafluoroethylene (PTFE) filters (25 mm, 0.5 *μ*m pore, SKC, Inc. catalog number 225-2708) were used for the collection stages. PTFE after-filters (37 mm, 2.0 *μ*m pore, SKC Inc., catalog number 225-1709) were used with each run. The pump's operating flow rate was calibrated by measuring volume displacement using a Defender Primary Standard Calibrator (SKC, Inc., catalog number 717-510H). A collection time of 41 min 36 sec was used to sample approximately 0.375 m^3^ of air. 

### 2.6. Positioning of Air Samplers

**Figure 2:**
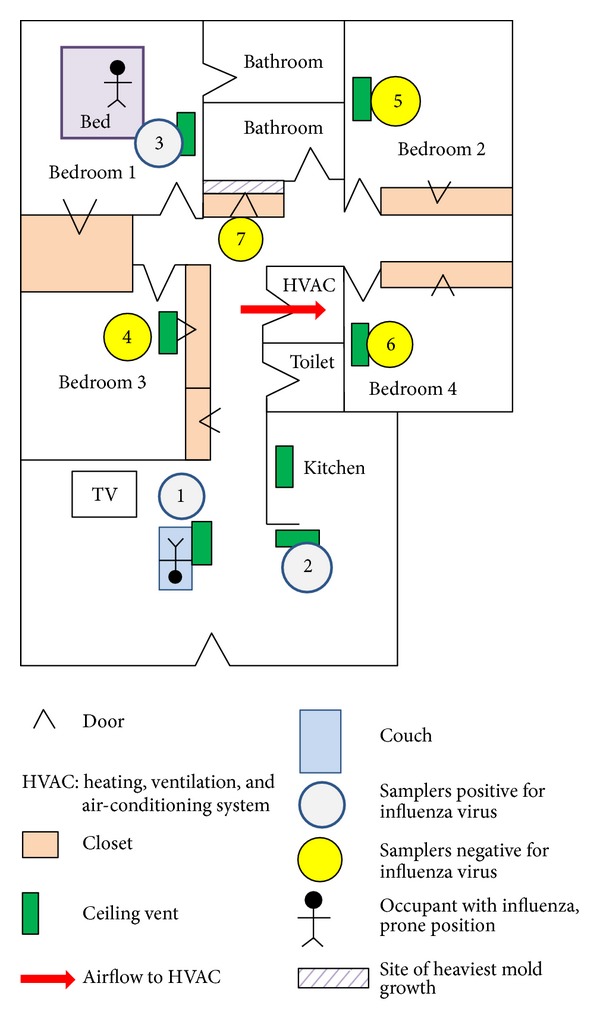
Schematic layout of the apartment unit of this study (drawn to scale). Symbols used for the diagram are explained in the figure legend.

PCIS and SKC BioSampler units were tandemly situated beneath ceiling vents so that air samplings would be made in areas with active air-flow (Figure 2). The room doors within the apartment were completely open during the air sampling period. The intake openings of the air samplers were at a height of 1 m and perpendicular to the ground, facing the patients. For comparisons of indoor to outdoor counts, outside air was evaluated using a PCIS and an SKC BioSampler in a courtyard outside the apartment. 

### 2.7. Influenza Diagnosis

The patients, an adult female (age 50) and young female (age 10), were diagnosed with influenza at a local clinic in early February 2012. Nasal discharges from both were positive for influenza A virus using a Binax NOW Flu A and Flu B test (Binax Inc., Portland, ME, USA). The kit did not distinguish between influenza A types H1 or H3. Both individuals exhibited classic signs of influenza, starting with a sudden onset fever (40°C), quickly followed by body ache, chills, dry cough, headache, malaise, muscle ache, pain on motion of the eyes, photophobia, red (and warm) flushed skin, sneezing, sore throat, watery eyes, and a feeling of severe weakness. Less characteristically, the adult but not the child suffered from diarrhea during the first day of illness. The adult had been vaccinated against influenza by injection, and the child by inhaled vaccine, the previous November (2012). Neither individuals had underlying health conditions and were otherwise healthy and physically fit.

### 2.8. Initial Workup of SKC BioSampler Virus Collection Media

Within 30 min of collection, virus collection media in the SKC BioSamplers was concentrated to a volume of about 400 *μ*L by ultrafiltration using Amicon Ultra 15 filter units with a molecular-weight cut off of 100 kD (Millipore, Bedford, MA, USA). The concentrated SKC BioSampler fluids were adjusted to a volume of 500 *μ*L by addition of PBS with 0.5%  w/v BSA fraction V, and were stored at −80°C.

### 2.9. Initial Workup of PCIS Filters for Virus Workup

The PCIS filters for virus tests were individually scraped with flocked swabs wetted with PBS with 0.5% w/v BSA fraction V then each swab eluted into separate 10 mL aliquots of PBS with 0.5% w/v BSA fraction V. The eluates were individually concentrated to a volume of about 400 *μ*L by ultrafiltration using Amicon Ultra 15 filter units, the volume adjusted to 500 *μ*L by the addition of sterile PBS with 0.5% w/v BSA fraction V and the concentrated PCIS fluids stored at −80°C. 

### 2.10. Cell Lines

MDCK (CCL-34) and Mv1 Lu (CCL-64) were obtained from the American Type Culture Collection (Manassas, VA, USA) and were propagated as monolayers at 37°C and 5% CO_2_ in Advanced Dulbecco's Modified Eagle's Medium (aDMEM) (Invitrogen Corp., Carlsbad, CA, USA) supplemented with 2 mM L-Alanyl-L-Glutamine (GlutaMAX, Invitrogen Corp.), antibiotics (PSN; 50 *μ*g/mL penicillin, 50 *μ*g/mL streptomycin, 100 *μ*g/mL neomycin (Invitrogen Corp.)), and 10% (v/v) low IgG, heat-inactivated gamma-irradiated fetal bovine serum (HyClone, Logan, Utah). MDCK-SIAT2,6-UF and Mv1 Lu-SIAT2,6-UF are cell lines prepared in this laboratory from the ATCC MDCK and Mv1 Lu cells and were propagated as described for ATCC cells. MDCK-SIAT2,6-UF and Mv1 Lu-SIAT2,6-UF overexpress influenza virus *α*2,6-linked sialic acid receptors [32]. Prior to use, all cell lines were treated for 3 weeks with plasmocin and verified free of mycoplasma DNA by PCR [32]. 

### 2.11. Virus Isolation

Aliquots (50 *μ*L) of concentrated fluids from the SKC BioSampler and PCIS filters were inoculated onto MDCK, Mv1 Lu [33], MDCK-SIAT2,6-UF, and Mv1 Lu-SIAT2,6UF in serum-free DMEM otherwise supplemented as previously described plus L-1-tosylamido-2-phenylethyl chloromethyl ketone (TPCK)-treated mycoplasma- and extraneous virus-free trypsin (Worthington Biochemical Company, Lakewood, NJ) in 5% CO_2_ at 33°C. The TPCK-trypsin was used at a final concentration 2 *μ*g/mL for MDCK and MDCK-SIAT2,6-UF cells and at 0.2 *μ*g/mL for Mv1 Lu-SIAT2,6-UF cells.

### 2.12. Rapid Detection of Influenza Virus in Cell Cultures

A commercial solid phase ELISA test (QuickVue influenza A and B kit, Quidel Corp., San Diego, CA, USA) was used to quickly detect influenza virus in cell cultures. The test did not distinguish between influenza A virus types H1 and H3.

### 2.13. Identification of Influenza A Virus Type by RT-PCR

Viral RNA was extracted from Mv1 Lu cells using a QIAamp Viral RNA Mini Kit, and RT-PCR was performed using primers described in a WHO Manual for influenza Virus Diagnostics [34]. Briefly, vRNA was denatured for 5 min at 67°C in the presence of SUPERase-In RNase inhibitor (Invitrogen Corp.), cooled rapidly, and in separate reactions, first strand synthesis was performed with Omniscript reverse transcriptase (RT) (Qiagen, Inc.) for 1 hr at 37°C with primers H1F1 (5′-AGCAAAAGCAGGGGAAAATAAAAGC-3′) for influenza A(H1N1) 2009 virus, THAF2 (5′-GCAGGGGAAAATAAAAACAACC-3′) for former seasonal influenza A(H1N1), and H3A1F6 (5′-AAGCAGGGGATAATTCTATTAACC-3′) for influenza A(H3N2). PCR was performed using One Taq DNA polymerase (New England Biolabs, Ipswich, MA. USA). Primers H1F1 and H1R1264 (5′-CCTACTGCTGTGAACTGTGTATTC-3′) were used for influenza A(H1N1) 2009 virus, with an expected product size of 1,264 bp, THAF2, and SPHAR11 (5′-TATTTTGGGCACTCTCCTATTG-3′) for former seasonal influenza A(H1N1), with an expected product size of 990 bp, and H3A1F6 and H3A1R1 (5′-GTCTATCATTCCCTCCCAACCAT-3′) for influenza A(H3N2), with an expected product size of 1,127 bp. PCR was performed as initial denaturation step; 94°C (30 sec); 30 cycles of 94°C (30 sec), 46°C (60 sec), and 68°C (1 min 25 sec); terminal extension step at 68°C (5 min); 4°C ∞. Gel electrophoresis (1.5% ethidium bromide-stained agarose gel) was used to visualize the PCR product.

### 2.14. Detection of Influenza Virus RNA in Collected Specimens

RNA was extracted from aliquots of concentrated SKC BioSampler and PCIS fluids using a QIAamp Viral RNA mini kit then subjected to RT-PCR analyses. Reverse transcription was performed with Omniscript RT using primer H3A1F6, and PCR was performed as described above with primers H3A1F6 and H3A1R1 to generate a 1127 bp amplification product.

### 2.15. Sequencing

The consensus sequences of the *hemagglutinin* and *neuraminidase* genes of the virus isolated in this work (designated A/Gainesville/07/2013 (H3N2)) were determined from PCR amplicons. The vRNA was reverse transcribed using AccuScript High Fidelity Reverse Transcriptase (Agilent Technologies, Inc., Santa Clara, CA) in the presence of SUPERase-In RNase inhibitor. PCR was performed using Phusion Polymerase (New England Biolabs) with denaturation steps performed at 98°C. A combination of primers mentioned in the WHO Manual for Influenza Virus Diagnostics [32] were used (information to be provided upon request). The PCR amplicons were purified using a Qiaquick PCR purification kit (Qiagen). Amplicon sequences were analyzed using an Applied Biosystem 3130 DNA analyzer by using BigDye Terminator (v. 3.1) chemistry and the same used for amplifications. 

### 2.16. GenBank

The *hemagglutinin* and *neuraminidase* gene sequences of influenza virus A/Gainesville/07/2013 (H3N2) were deposited in GenBank (accession numbers KF061021 and KF061022).

### 2.17. Plaque Assays

Plaque assays were performed as in [33], though MDCK (and not Mv1 Lu) cells were used so that our results would be easier to compare and contrast with those of others (since most laboratories use MDCK cells for influenza virus plaque assays). 

## 3. Results

The RH of indoor air at the residence ranged from 44 to 45%, and indoor *T*'s ranged from 21 to 23°C during the testing period of this work. 

Viable virus was successfully isolated from samplers located up to 3.7 m away from one of the sick occupants (Table 1). PCIS filters that yielded viable virus are given in Tables 2 and 3. Only samplers located at sites 1, 2, and 3, which were close to and in a direct path with sick individuals (Figure 2), collected RT-PCR detectable or viable virus. 

The PCIS collected a range of particle sizes containing influenza virus when it was 1.2 m away from the ill person resting on a couch (at site 1) but presumably only fine to ultrafine particles (≤2.5 *μ*m) at distances of 2.1 and 3.7 m away from the sick persons (Table 3). 

**Figure 3:**
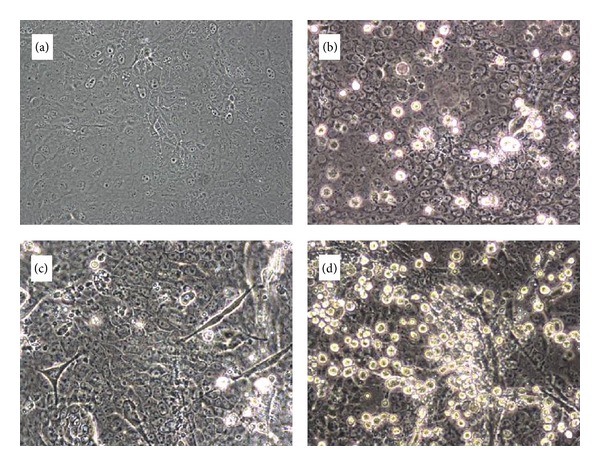
Early formation of influenza virus-specific CPE in cells inoculated with material collected by a PCIS at air sampling site 3. (a) Noninfected ATCC MDCK cells (negative control), 3 days postseed. (b) ATCC MDCK cells inoculated with material scraped off the PCIS after-filter. (c) Noninfected MDCK-SIAT2,6-UF cells, (negative control), 3 days postseed. (d) MDCK-SIAT2,6-UF cells inoculated with material scraped off the PCIS after-filter.

For each sample set, influenza virus-specific CPE were apparent earlier in MDCK-SIAT2,6-UF and Mv1 Lu-SIAT2,6-UF cells than in conventional MDCK and Mv1 Lu cells. An example is shown in Figure 3 for CPE (granulation of the cytoplasm, enlargement of nuclei, followed by rounding of the cells and detachment from the growing surface) three days postinfection of MDCK and MDCK-SIAT2,6-UF cells that had been inoculated with eluate from the after-filter of a PCIS positioned at site 3. However, for each sample wherein virus was isolated, CPE developed in all the cell types that were used by 6 days postinfection. A Quidel QuickVue Influenza A and B kit was used to quickly confirm the presence of virus in the cells. As observed by others [13, 14], virus was detected by RT-PCR in some samples wherein viable virus was not isolated (Table 3). 

**Figure 4:**
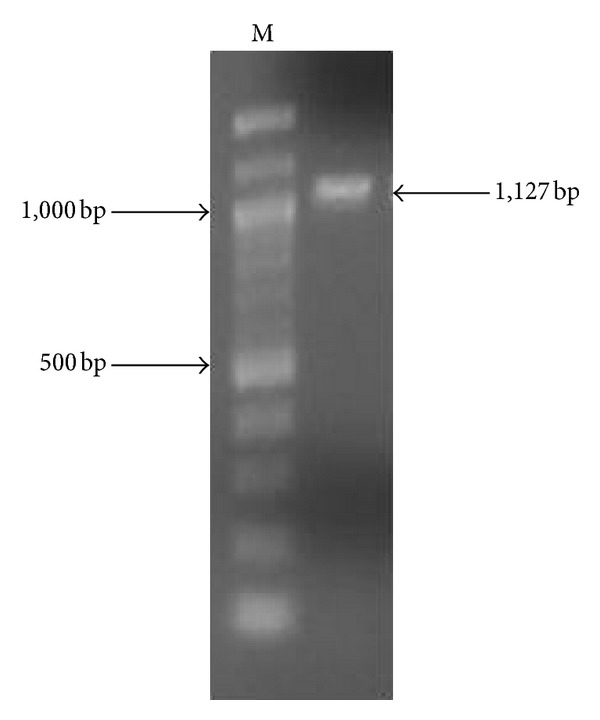
RT-PCR amplification of an influenza virus H3N2 subgenomic hemagglutinin gene sequence. Shown in an ethidium-bromide-stained 1.5% agarose gel loaded with a 100-bp ladder (lane marked “M”) and a 1,127 bp amplicon corresponding to the N-terminal half of the hemagglutinin gene sequence.

RT-PCR analyses indicated that influenza A(H3N2) had been isolated in cell culture, as primers for the detection of pandemic H1N1 and seasonal H1N1 virus did not amplify a specific product, whereas primers for H3N2 amplified the expected 1,127 bp amplicon corresponding to the anterior half of the hemagglutinin gene sequence (Figure 4). The virus that had been isolated was different from strains present in the laboratory, ruling out laboratory contamination. A simple BLAST homology search against sequences in GenBank as of 27 May 2013 indicated that the HA gene of influenza virus A/Gainesville/07/2013 (H3N2) was most similar to that of influenza virus (A/Washington/3262/2012(H3N2)). 

## 4. Discussion

The RH within the apartment (44-45%) was within the optimal comfort level for humans (40–60%), and the interior *T* was also comfortable. Various studies indicate that aerosol transmission of influenza virus is decreased at an RH greater than about 50%, and that transmission of airborne influenza virus is best under cold and dry conditions ([35] and references therein). Since the RH of the apartment was 44-45%, and the *T* was between 22 and 23°C, we predicted that viable airborne virus would be detected, and this turned out to be the case.

Viable “live” virus was present in the liquid collection media of the SKC BioSampler, and some but not all of the filters of the PCIS (Table 3). Prior to drawing conclusions, modeling is needed with *in vitro* generated influenza virus aerosols to determine if the results of Table 3 truly reflect a difference in collection efficiency between the SKC BioSampler and the PCIS. It is possible that a large proportion of the collected airborne virus was damaged during impaction with the PTFE filters, yet the actual airborne virus collection efficiency of the PCIS was higher than that of the SKC BioSampler. Alternatively, it is also possible that under a constantly flowing airstream during active sampling with a PCIS, that influenza virus collected on the PTFE filters gets desiccated (and thus inactivated) on the filters [23]. 

Whereas it is already acknowledged that influenza patients expel large droplets as they cough and sneeze, our findings suggest that influenza patients also produce small aerosol particles (≤5 *μ*m) that contain viable virus. Viable virus was detected in areas close to the two patients but not in adjoining rooms separated by curved paths. Because fluid dynamics modeling was not used to study air-circulation in this work, it was not clear whether positioning of the samplers in a direct path to sick individuals was important; perhaps the overriding factor was closeness to the persons.

 The *hemagglutinin* gene of influenza virus A/Gainesville/07/2013(H3N2) is essentially identical to that of A/Washington/3262/2012(H3N2) (GenBank Accession number CY141277). It has less sequence homology to the corresponding gene of A/Gainesville/01/2012(H3N2) (GenBank Accession number KF142471), which circulated in Gainesville in 2012. Since Florida and Washington are situated on opposite coasts of the USA, it is plausible that A/Gainesville/07/2013(H3N2) stemmed from someone who had traveled to Florida from the West coast. Sequence analyses of the other genes of A/Gainesville/07/2013(H3N2) indicated that the virus was similar to other nonvariant H3N2 strains in circulation in the USA, suggesting influenza vaccines were not protective for the two patients of this report.

These findings of this work are consistent with recent reports of influenza viruses in fine particle aerosols in the exhaled breath of influenza patients [13, 14], and collectively, these reports challenge the notion that influenza virus is only transmitted through mucous contact with large droplets. 

## Figures and Tables

**Table 1:** Location of air sampler sites yielding viable virus.

	Location of air samplers and isolation of influenza H3N2 virus in cell cultures						
	Site 1	Site 2	Site 3	Site 4	Site 5	Site 6	Site 7
SKC BioSampler	+^b^	+	+	−^c^	−	−	−
PCIS	+	+	+	−	−	−	−
Distance from closest sick person^a^	1.2 m	2.1 m	3.7 m	>4 m	>4 m	>4 m	>4 m
Pathway from closest sick person	Direct	Direct	Direct	Curved	Angled	Angled	Angled

^a^Approximate distance from head of resting (laying down) person.

^b^Virus isolated in cell cultures.

^c^Virus not isolated in cell cultures.

**Table 2:** Detection and isolation of influenza H3N2 virus in concentrated virus collection media.

		RT-PCR detection and virus isolation						
		Site 1	Site 2	Site 3	Site 4	Site 5	Site 6	Site 7
SKC BioSampler		D^a^, VI^b^	D, VI	D, VI	—, —	—, —	—, —	—, —
PCIS								
Filter position	Cutoff size							
Stage A	>2.5 *μ*m	D, VI	—, —	—, —	—, —	—, —	—, —	—, —
Stage B	1.0–2.5 *μ*m	D, —	—, —	—, —	—, —	—, —	—, —	—, —
Stage C	0.5–1.0 *μ*m	D, VI	D, VI	D, VI	—, —	—, —	—, —	—, —
Stage D	0.25–0.5 *μ*m	D, VI	D, VI	D, VI	—, —	—, —	—, —	—, —
After-filter	<0.25 *μ*m	D, VI	D, —	D, VI	—, —	—, —	—, —	—, —

^a^D: detection of viral RNA by RT-PCR.

^b^VI: virus isolation.

**Table 3:** Approximate viral count in concentrated SKC BioSampler and PCIS collection media.

		Approximate no. of viable virions^a^		
		Site 1	Site 2	Site 3
SKC BioSampler		~2,500	~130	~1,200
PCIS				
Filter position	Cut-off size			
Stage A	>2.5 *μ*m	~60	—	—
Stage B	1.0–2.5 *μ*m	—	—	—
Stage C	0.5–1.0 *μ*m	~50	~30	~20
Stage D	0.25–0.5 *μ*m	~390	~70	~180
After-filter	<0.25 *μ*m	~130	—	~110

^a^Approximate (total) number of viable virions in 500 *μ*L of concentrated collection media (from 0.375 m^3^ of sampled air).
